# Role of fecal calprotectin as a hypoxic intestinal damage biomarker in COVID-19 patients

**DOI:** 10.1186/s13099-022-00507-y

**Published:** 2022-08-09

**Authors:** Deasy Natalia Adriana, Titong Sugihartono, Iswan Abbas Nusi, Poernomo Boedi Setiawan, Herry Purbayu, Ummi Maimunah, Ulfa Kholili, Budi Widodo, Husin Thamrin, Amie Vidyani, Hasan Maulahela, Yoshio Yamaoka, Muhammad Miftahussurur

**Affiliations:** 1 Department of Internal Medicine, Faculty of Medicine, Soetomo Teaching Hospital, Universitas Airlangga, Dr, Surabaya, 60286 Indonesia; 2 Gastroenterology and Hepatology Division, Department of Internal Medicine, Faculty of Medicine, Dr. Soetomo Teaching Hospital, Universitas Airlangga, Surabaya, 60286 Indonesia; 3 Division of Gastroenterology, Department of Internal Medicine, Faculty of Medicine, University of Indonesia, Jakarta, Indonesia; 4 Helicobacter Pylori and Microbiota Study Group, Institute of Tropical Disease, Universitas Airlangga, Surabaya, 60115 Indonesia; 5 Department of Environmental and Preventive Medicine, Faculty of Medicine, Oita University, 1-1 Idaigaoka, Hasama-machi, Yufu, Oita 879-5593 Japan; 6 Department of Medicine, Gastroenterology and Hepatology Section, Baylor College of Medicine, Houston, TX USA

**Keywords:** Fecal calprotectin, P/F ratio, COVID-19, Infectious disease

## Abstract

**Background:**

Gastrointestinal manifestations of coronavirus disease 2019 (COVID-19) appear to be substantial. Fecal calprotectin is a promising biomarker in COVID-19 associated gastrointestinal inflammation; however, its role in the severity of COVID-19 remains limited. We conducted a study to analyze the relationship between the severity of COVID-19 and hypoxic intestinal damage.

**Methods:**

We assessed the severity of 44 hospitalized COVID-19 pneumonia patients based on the PaO2/FiO2 (P/F) ratio. Inflammatory markers were measured from blood samples, and fecal calprotectin was obtained from stool samples.

**Results:**

Median levels of fecal calprotectin in COVID-19 patients involved in this study (n = 44) were found to be markedly elevated along with the severity of hypoxemia, as seen in the non-acute respiratory distress syndrome (ARDS) group 21.4 µg/g (5.2–120.9), mild ARDS 54.30 µg/g (5.2–1393.7), moderate ARDS 169.6 µg/g (43.4–640.5), and severe ARDS 451.6 µg/g (364.5–538.6). We also found significant differences in fecal calprotectin levels based on the severity of ARDS (*P* < 0.001), and although the patients were divided into ARDS and non-ARDS groups (*P* < 0.001). Furthermore, we found a strong negative correlation between the P/F ratio and fecal calprotectin levels (*r* = − 0.697, *P* < 0.001).

**Conclusion:**

Our findings support the potential role of fecal calprotectin as a biomarker of intestinal inflammation in COVID-19 as a consequence of hypoxic intestinal damage and as suggested by the reduced P/F ratio.

## Background

Coronavirus disease 2019 (COVID-19) is an infectious disease caused by the severe acute respiratory syndrome coronavirus (SARS-CoV-2). It was declared a pandemic in March 2020 and currently remains a global health issue [1–3]. COVID-19 presents with a wide spectrum of clinical conditions, ranging from an asymptomatic course to severe pneumonia, which can lead to acute respiratory distress syndrome (ARDS) marked by a decreased PaO2/FiO2 (P/F) ratio. Apart from respiratory diseases, COVID-19 can also cause extrapulmonary manifestations, including gastrointestinal symptoms [4–8]. Gastrointestinal symptoms, such as anorexia, nausea, vomiting, and abdominal pain, are commonly found in approximately 20–50% of COVID-19 cases; nonetheless, intestinal injury might occur even without the presence of gastrointestinal symptoms [9–13].

The etiology of gastrointestinal injury in COVID-19 can be both primary and secondary. Primary injury occurs due to direct infection of SARS-CoV-2 in the gastrointestinal system, whereas secondary injury arises from several conditions [14–18]. The injury might be related to hypoxemia, marked by a decreased P/F ratio, which leads to hypoxic intestinal damage. Injury might also occur as a result of a dysregulated immune response, also known as cytokine release syndrome, which causes systemic inflammation [19–22]. SARS-CoV-2 infection can also lead to hypercoagulability and microcirculatory dysfunction, which in turn leads to ischemia in tissues, including the gastrointestinal system [23, 24]. On the other hand, the inflammation process in the gastrointestinal tract may also worsen the ongoing systemic inflammation by a mechanism known as intestinal crosstalk [25]. This inflammation might be observed during the endoscopic procedure, whose performance is limited during the COVID-19 pandemic due to safety issues [26].

Due to its stability in the stool for 5–7 days, fecal calprotectin is a sensitive and noninvasive biomarker of intestinal inflammation [27, 28]. Fecal calprotectin is a calcium-binding and primarily neutrophil-specific protein that is released into the extracellular environment as a result of a neutrophil disintegration cascade during acute inflammation. Interestingly, the concentration of fecal calprotectin, which accounts for 60% of the cytosolic protein in neutrophils, is proportional to the concentration of neutrophils in the intestinal mucosa, wherein the functions are severely affected by ischemia [26, 29, 30]. Therefore, fecal calprotectin is a potential biomarker for hypoxic intestinal damage in COVID-19 patients.

Studies evaluating intestinal inflammation in COVID-19 patients using fecal calprotectin levels are still limited. Only six studies and one meta-analysis evaluated the subjects [23, 31–36]. Among these studies, only three investigated the relationship between the severity of COVID-19 and intestinal inflammation. These three studies used various definitions of disease severity with contradictory results [31, 34, 35]. A study in Italy demonstrated a significant correlation between elevated fecal calprotectin levels and COVID-19 disease severity, characterized by the presentation of pneumonia [34]. Another study in Iran also suggested that elevated fecal calprotectin levels may be a feature of severe disease, with a significant positive association between disease severity and fecal calprotectin levels [35]. In contrast, the first fecal calprotectin in the COVID-19 study launched in the United States indicated no correlation between the concentration of fecal calprotectin and COVID-19 disease severity [31].

Therefore, we analyzed the association between COVID-19 disease severity based on the degree of hypoxemia using the P/F ratio and intestinal inflammation caused by hypoxic intestinal damage, which is determined by fecal calprotectin levels. We applied the P/F ratio as the measurement of COVID-19 disease severity by taking into account the oxygen fraction given to the patients to standardize the degree of hypoxemia in patients with and without oxygen supplementation. In addition, we observed the characteristics of gastrointestinal manifestations, general symptoms, and inflammatory markers in COVID-19 patients in the Indonesian population.

## Methods

### Study design and participant

This was an observational analytical study using a cross-sectional approach. We analyzed the P/F ratio from blood gas analysis and fecal calprotectin from stool samples of 44 patients confirmed COVID-19 based on positive nasopharyngeal SARS-CoV-2 PCR swabs with suggestive COVID-19 radiological appearances [2, 37]. We included all hospitalized patients from non-ICU COVID-19 isolation units at Dr. Soetomo Teaching Hospital, Surabaya, Indonesia from October to December 2020 who fulfilled the inclusion criteria. The exclusion criteria involved those patients with gastrointestinal malignancies, inflammatory bowel disease, cirrhosis, and end-stage renal disease. Written informed consent was obtained from all patients, and the study protocol was approved by the ethics committee of the Dr. Soetomo Teaching Hospital Surabaya, Indonesia (0065/KEPK/IX/2020). We declare that all procedures contributing to this research comply with the ethical standards of the relevant national and institutional committees on human experimentation and the Helsinki Declaration of 1975 (as revised in 2008 and 2013).

### COVID-19 disease severity

We obtained the P/F ratio from blood gas analysis, which was performed within 24 h of the collection of stool samples used for fecal calprotectin testing. Blood gas from arterial samples was sent to the laboratory within 15 min or stored in 0-4^O^C. The samples were then analyzed using GEM Premier®. We used the P/F ratio to express COVID-19 disease severity and standardized the degree of hypoxemia in subjects with and without supplemental oxygen, thus representing the severity of COVID-19 [38, 39]. We only included subjects with radiologic appearances suggestive of COVID-19 to minimize the possibility of a decreased P/F ratio due to etiologies other than COVID-19 ARDS. The P/F ratio (mmHg) was then divided into categories based on the Berlin criteria of ARDS; mild ARDS was defined as a threshold of 200 < P/F ≤ 300, moderate ARDS 100 < P/F ≤ 200, and severe ARDS ≤ 100 [40].

### Fecal calprotectin

Stool samples were collected into clean containers with a minimum amount of 5 g, registered, and stored at 2–8 °C for 48 h or − 20 °C for more than 48 h, and then used for fecal calprotectin measurement. Fifteen milligrams of stool from each sample were extracted and analyzed using the PhiCal^©^ Calprotectin enzyme-linked immunosorbent assay (ELISA) kit according to the manufacturer’s recommendations (Immundiagnostik AG, Stubenwald-Allee 8a, D-64625 Bensheim). Calprotectin samples remained stable in the stool for 5–7 days with a reference normal value of < 50 μg/g [27, 28].

### Additional laboratory measurements

All other laboratory parameters were obtained within 24 h of stool sample collection and were determined in the hospital laboratory as part of the routine laboratory analysis. Samples from the complete blood count, such as leukocytes (/µL), thrombocytes (/µL), and neutrophil-to-lymphocyte ratio (NLR) were analyzed using Sysmex 1000®. Ferritin (ng/mL) was analyzed using a two-site sandwich immunoassay with direct chemiluminometric technology using ADVIA Centaur Ferritin®. D-dimer (ng/mL) was obtained from serum samples and analyzed using a turbidimetric immunoassay with Sysmex CS-2500®. C-reactive protein (CRP) was analyzed by a particle-enhanced turbidimetric immunoassay using the Dimension® Clinical Chemistry System and was defined in mg/L.

### Statistical analysis

Statistical analysis was performed using IBM SPSS® Statistics version 25 (IBM Corp., USA). Demographic data and clinical characteristics are presented descriptively by frequency and percentage for categorical data types (nominal and ordinal). Continuous data (interval and ratio) are shown as mean ± standard deviation (SD) and median (minimum–maximum). Normality tests were carried out using the Shapiro–Wilk test. The independent variable was the P/F ratio, which was presented as ordinal and ratio data. The dependent variable was fecal calprotectin level, presented as nominal and ratio data. Independent t-tests were used to compare normally distributed data, whereas Mann–Whitney and Kruskal–Wallis tests were used for the analysis of non-normal data. Analysis of the association between variables in this study was carried out by correlational numerical analysis using Spearman correlation. *P*-value < 0.05 was considered statistically significant with a confidence interval (CI) of 95%.

## Results

### Patient demographic and clinical characteristics

In the present study of 44 hospitalized COVID-19 patients with suggestive radiologic appearance, 26 patients were negative for fecal calprotectin (< 50 µg/g), and 18 patients were positive for fecal calprotectin (≥ 50 µg/g). As shown in Table 1, there was no significant difference in sex between the fecal calprotectin positive (FC-positive) and negative (FC-negative) groups (*P* = 0.241). The mean age in the FC-positive group was slightly higher than those in the FC-negative group (49.9 ± 14.8 vs 47.8 ± 13.9 years); nevertheless, there was no significant difference in age between the two groups (*P* = 0.573). Most of the patients (59.1%) had comorbidities, and diabetes mellitus was the major comorbidity in all groups (total population, FC-positive, and FC-negative groups).

**Table 1:** Baseline characteristics of hospitalized patients with COVID-19 stratified by fecal calprotectin level

Characteristics	Total (n = 44)	FC-negative (< 50) (n = 26)	FC-positive (≥ 50) (n = 18)	*P-*value
Gender				0.241
Male	23 (52.3%)	16 (61.5%)	7 (38.9%)	
Female	21 (47.7%)	10 (38.5%)	11 (61.1%)	
Age (years)	48.7 ± 14.2	47.8 ± 13.9	49.9 ± 14.8	0.573
18–59	32 (72.7%)	19 (73.1%)	13 (72.2%)	
≥ 60	12 (27.3%)	7 (26.9%)	5 (27.8%)	
Any comorbidity*	26 (59.1%)	16 (61.5%)	10 (55.6%)	0.932
Hypertension	18 (40.9%)	12 (46.2%)	6 (33.3%)	0.590
Diabetes Mellitus	22 (50%)	13 (50%)	9 (50%)	1.000
Respiratory symptoms*				
Fever	31 (70.5%)	18 (69.2%)	13 (72.2%)	1.000
Cough	33 (75%)	19 (73.1%)	14 (77.8%)	1.000
Dyspnea	30 (68.2%)	18 (69.2%)	12 (66.7%)	1.000
Anosmia	4 (9.1%)	3 (11.5%)	1 (5.6%)	0.634
Gastrointestinal symptoms*				
Nausea	17 (38.6%)	1 (3.8%)	16 (88.9%)	** < 0.001**
Vomiting	13 (29.5%)	0 (0%)	13 (72.2%)	** < 0.001**
Diarrhea	14 (31.8%)	1 (3.8%)	13 (72.2%)	** < 0.001**
Abdominal pain	5 (11.4%)	1 (3.8%)	4 (22.2%)	0.142
Decreased appetite	28 (63.6%)	13 (50%)	15 (83.3%)	0.052
Laboratory findings				
Leukocyte (/µL)	10 113.4 ± 4471.21	9465.8 ± 2802.4	11 048.9 ± 6119.6	0.775
Thrombocyte (/µL)	335 227.3 ± 135 671.3	345 884.6 ± 135 865.9	319 833.3 ± 137 793.6	0.537
NLR	7.5 ± 8.2	6.3 ± 3.3	9.33 ± 12.12	0.567
Ferritin (ng/ml)	761.3 ± 664.3	774.5 ± 669.6	739.8 ± 676.8	0.736
D-Dimer (ng/ml)	4469.4 ± 8327.3	4536.7 ± 8048.8	4372.2 ± 8950.5	0.384
CRP (mg/L)	1.9 ± 2.4	1.6 ± 1.7	2.6 ± 3.2	0.952

^*^Each patient might have more than one comorbidity or symptom. *NLR* neutrophil-to-leukocyte ratio, *CRP* C-reactive protein. Data are presented as numbers (percentages) or mean ± standard deviation (SD). Bold font indicates statistical significance at the *P*-value < 0.05

Decreased appetite was the most commonly reported gastrointestinal symptom in this study (63.6%, 28/44). Interestingly, the frequency of all features of gastrointestinal symptoms was higher in the FC-positive group than in the FC-negative group, and statistically significant differences between the two distinct groups were noted for nausea (*P* < 0.001), vomiting (*P* < 0.001), and diarrhea (*P* < 0.001). Coughing was the major complaint in all groups, followed by fever, dyspnea, and anosmia. No notable differences were found between the FC-positive and FC-negative groups for any of the measured systemic inflammatory markers (NLR, ferritin, CRP, and D-dimer).

### Elevated fecal calprotectin level is associated with severity of hypoxemia

**Fig. 1:**
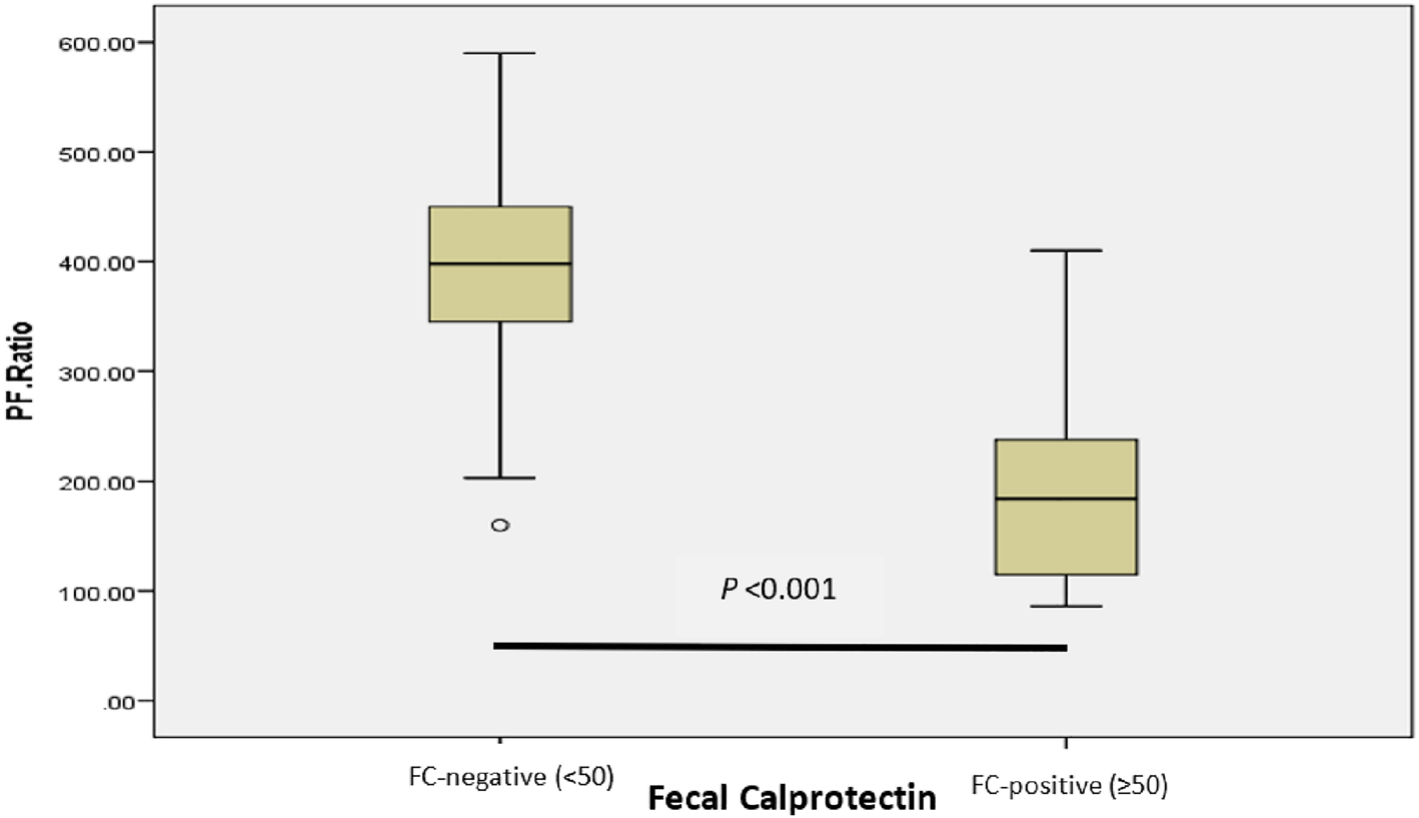
Comparison of P/F ratio (mmHg) between FC-negative and FC-positive groups (µg/g) by independent t-test

Importantly, most subjects were found to have ARDS (33.3% mild, 6/18; 50% moderate, 9/18; 11.11% severe, 2/18) in the FC-positive group as compared to the FC-negative group, wherein only 19.23% (n = 5/26) were diagnosed with ARDS (Table 2). The mean P/F ratio of the FC-positive group was also significantly lower than that of the FC-negative group (190.83 ± 82.41 mmHg vs 396.19 ± 100.45 mmHg), as illustrated in Fig. 1.

**Table 2:** The proportion of patients based on P/F ratio within fecal calprotectin groups

P/F Ratio (mmHg)	Total (n = 44)	FC-negative (< 50) (n = 26)	FC-positive (≥ 50) (n = 18)
Mean ± SD	312.18 ± 137.78	396.19 ± 100.45	190.83 ± 82.41
P/F Ratio ≤ 100 (Severe ARDS)	2 (4.54%)	0 (0%)	2 (11.11)
100 < P/F Ratio ≤ 200 (Moderate ARDS)	10 (22.72%)	1 (3.85%)	9 (50%)
200 < P/F Ratio ≤ 300 (Mild ARDS)	10 (22.72%)	4 (15.39%)	6 (33.33%)
*P/F Ratio* > 300 (Non-ARDS)	22 (50%)	21 (80.77%)	1 (5.56%)

^*^Data are presented as numbers (percentages) and mean ± SD. P/F, PaO2/ FiO2; *ARDS* acute respiratory distress syndrome, *FC* fecal calprotectin

**Fig. 2:**
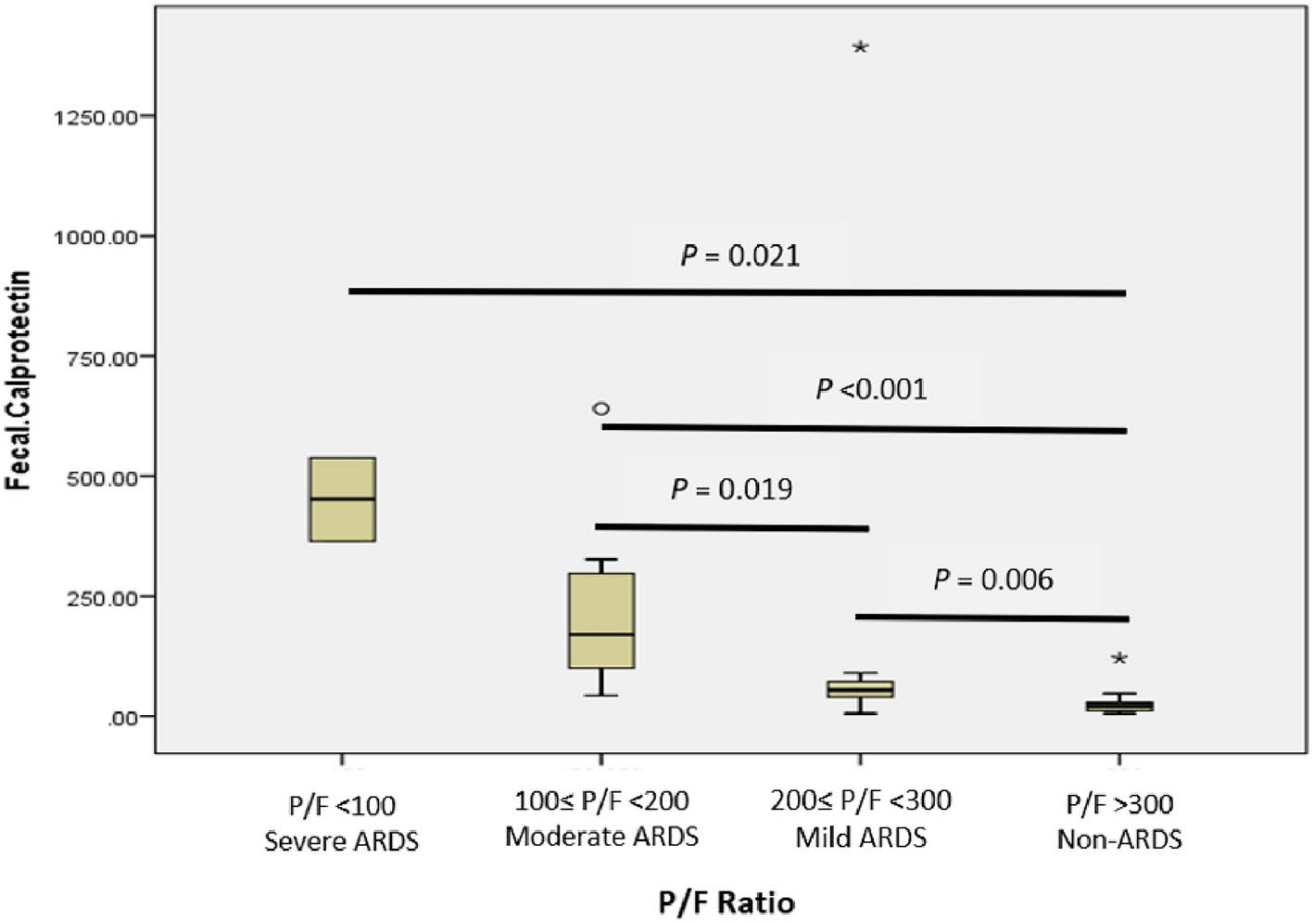
Concentration of fecal calprotectin (µg/g) stratified by severity of hypoxemia (P/F ratio in mmHg)

The analysis of fecal calprotectin level in COVID-19 patients stratified by the degree of hypoxemia showed that median fecal calprotectin levels were elevated along with the severity of ARDS as seen in non-ARDS group 21.35 µg/g (5.20–120.90), mild ARDS 54.30 µg/g (5.20–1393.70), moderate ARDS 169.55 µg/g (43.40–640.50), and severe ARDS 451.55 µg/g (364.50–538.60). Kruskal–Wallis analysis also showed a statistically significant difference in fecal calprotectin levels stratified by hypoxemia severity (*P* < 0.001). We further analysed the pairwise differences between the groups and discovered significant differences in individual ARDS groups relative to the non-ARDS group, as shown in Fig. 2.

**Fig. 3:**
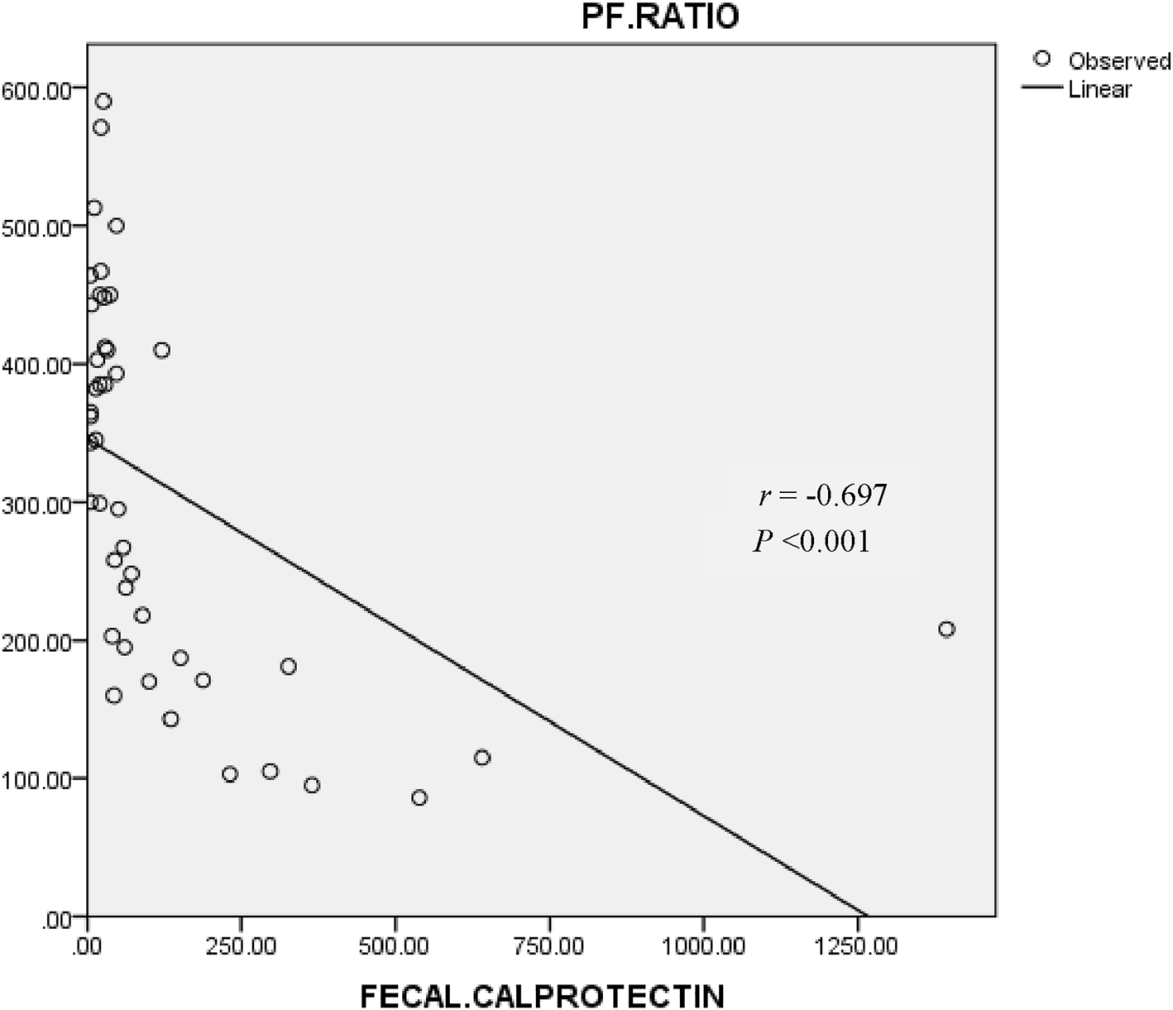
The relationship of P/F ratio (mmHg) and fecal calprotectin level (µg/g) analyzed by Spearman correlation

Considering the hypothesis in the present study that disease severity based on the degree of hypoxemia may be related to intestinal inflammation measured by fecal calprotectin, we established a correlation analysis. Spearman’s correlation, as seen in Fig. 3, revealed a strong negative correlation between the P/F ratio and fecal calprotectin level (*r* = -0.697, *P* < 0.001). This result revealed the relationship between P/F ratio and fecal calprotectin, leading to the statistical conclusion that deteriorated intestinal inflammation presented by elevated fecal calprotectin level was consistent with worsened hypoxemia measured by reduced P/F ratio.

## Discussion

To the best of our knowledge, this is the first study to investigate the relationship between the P/F ratio and fecal calprotectin in COVID-19. Fecal calprotectin levels were significantly increased in patients with COVID-19, along with worsened hypoxemia. The distributions of fecal calprotectin levels in different groups of patients with ARDS were also significantly different from those in their non-ARDS counterparts. A strong correlation between the P/F ratio and fecal calprotectin discovered in this study also highlighted the relationship between disease severity and intestinal inflammation due to hypoxic intestinal damage in COVID-19 patients. Fecal calprotectin is a neutrophil-specific protein whose role is greatly impacted by intestinal ischemia [23, 30]. These results were consistent with a previous study in Italy that found a significant relationship between COVID-19 pneumonia and fecal calprotectin levels in COVID-19, wherein pneumonia represented disease severity [34].

Interestingly, despite the significant association between the P/F ratio and fecal calprotectin level, our results revealed statistically significant differences between each degree of ARDS compared to the non-ARDS group and between the ARDS and non-ARDS groups. These results show us that fecal calprotectin's role in representing intestinal inflammation can be viewed primarily by comparison in conditions with and without hypoxemia but is less prominent in between the degrees of hypoxemia. This particular finding brings us to the theory of hypoxia and mucosal inflammation, where hypoxia-inducible factor (HIF) might play an important role. HIF is a key regulator of inflammatory hypoxia in the intestine in regard to inflammatory resolution [41]. Stimulation of HIF-1α in the intestine generates a barrier-protective pathway by enhancing mucus, defensin, and tight junction proteins, as well as refilling the ATP pool at the time of injury. HIF also regulates erythropoiesis, angiogenesis, and metabolism in the intestine as an adaptation to hypoxia [42]. This adaptation leads to inflammatory resolution, as seen in less affected fecal calprotectin levels (no statistically significant differences between degrees of ARDS).

The main gastrointestinal symptom in this study was decreased appetite, except in the FC-positive group wherein nausea became the leading symptom. This finding is parallel to a systematic review and meta-analysis on the prevalence of gastrointestinal symptoms from 78 studies with 12,797 COVID-19 patients wherein loss of appetite was the most prevalent (approximately one-fifth of patients) [43]. In contrast to two previous studies, nausea, vomiting, and diarrhea in our study showed striking statistical differences between the FC-positive group and the FC-negative group [31, 35]. In this regard, a previous study in Austria stated that SARS-CoV-2 infection induces an inflammatory response in the intestine, as indicated by diarrhea and elevated fecal calprotectin [33]. Nonetheless, since we did not evaluate SARS-CoV-2 PCR from fecal samples, it is premature to determine whether diarrhea and other gastrointestinal symptoms in the current study developed from direct viral etiology or due to other inflammatory processes in the intestinal mucosa.

Another notable finding is that no statistically significant differences between FC-positive and FC-negative groups for all inflammatory parameters were found in this current study. In this regard, a previous study found a significant correlation between fecal calprotectin and serum IL-6 concentration (*P* < 0.001), but not CRP or ferritin [33]. The results from this previous study in Austria may support the hypothesis that SARS-CoV-2 induces gastrointestinal inflammation without direct invasion of intestinal cells. Circulating inflammatory cytokines can induce cellular infiltration of the intestinal wall, which in turn leads to calprotectin release [35, 44]. Nonetheless, our results showed that the role of this particular mechanism was less significant in our study population. In contrast to another previous study from Italy, we found no significant difference in D-dimer levels between the fecal calprotectin groups [23]. This result suggests that the role of hypercoagulability in triggering intestinal inflammation in our subjects was subtle.

This study had several limitations. First, the sample size was relatively small, since this was a single-center study; hence, the results should be validated with additional studies with larger sample sizes and multicenter studies if possible. Second, this was a cross-sectional study, not a prospective study. It is difficult to determine the direction of the relationship between the P/F ratio and fecal calprotectin using this approach since both variables can interfere with each other. A prospective study may also allow us to evaluate the trend of fecal calprotectin levels throughout the hospitalization period and to determine the outcome. We also did not have data regarding the onset of symptoms and the time of hospitalization to blood and stool sample collection, which might provide information on the relationship between COVID-19 clinical courses and intestinal inflammation. Finally, we did not evaluate for SARS-CoV-2 PCR in fecal samples; thus, we could not eliminate whether there was direct viral invasion of the intestinal mucosa, instigating intestinal inflammation. Nevertheless, this study is valuable as a preliminary study to reinforce further research in this field.

## Conclusion

In summary, our findings support the current understanding of the relationship between the severity of COVID-19 and intestinal manifestations. Fecal calprotectin shows a potential role as a biomarker of intestinal inflammation in COVID-19 as a consequence of hypoxic intestinal damage, as suggested by the reduced P/F ratio. Nonetheless, more studies are required to investigate the etiology of gastrointestinal manifestations and elevated fecal calprotectin levels in patients with COVID-19, along with their potential in predicting gastrointestinal complications and clinical outcomes.

## Data Availability

All sequences and associated metadata for the current study are available from the corresponding author upon reasonable request. On behalf of research collaboration and development, the patients and the ethics committee were allowed to share the data with the corresponding authors' consideration.
